# A profile of substance abusers using the emergency services in a tertiary care hospital in Sikkim

**DOI:** 10.4103/0019-5545.31556

**Published:** 2006

**Authors:** Akhil Bhalla, Sanjiba Dutta, Amit Chakrabarti

**Affiliations:** * Student, MBBS, Semester VII Sikkim Manipal Institute of Medical Sciences, 5th Mile, Tadong, Gangtok 737102, Sikkim; ** Associate Professor of Psychiatry Sikkim Manipal Institute of Medical Sciences, 5th Mile, Tadong, Gangtok 737102, Sikkim; *** Additional Professor of Pharmacology and Epidemiology Sikkim Manipal Institute of Medical Sciences, 5th Mile, Tadong, Gangtok 737102, Sikkim

**Keywords:** Substance abuse, emergency service, alcohol, Sikkim

## Abstract

**Background:**

Sikkim, a state in Northeast India with a population of more than 500,000 and inhabited by indigenous population of Lepchas, Bhutias and Nepalis, lies in the foothills of the Himalayas sharing borders with Tibet, Nepal and Bhutan. Northeast India is a major source of injection drug users (IDUs) and associated HIV/AIDS. Alcohol use is traditionally prevalent in Sikkim and recently, IDU behaviour has also been reported, although systematic information on epidemiology and treatment availability of substance abuse in Sikkim is not available.

**Aim:**

To study the sociodemographic and drug use profile of substance abusers using the emergency services in a tertiary care hospital.

**Methods:**

A retrospective chart review was used. Patients with history of current drug use seeking emergency services for any medical or surgical consequence incident to substance abuse from July 2000 to June 2005 (60 months) were included in the study. Data were generated from emergency case register, hospital records and case sheets. SPSS 10.0 was used for data analysis.

**Results:**

Out of 54 patients seeking emergency services with substance abuse (1.16% of all psychiatric consultations), alcohol abusers were 77.8% and other opioid abusers 14.8%. Prevalence of IDU was 16.66%. Common opioids abused were dextrpropoxyphene and pentazocine, both analgesics. A significant number of patients (46.3%) had a history of >20 days/month frequency of abuse. Median of duration of abuse with all drugs was 12 years, while that with IDU population was 3 years. Alcohol withdrawal was the commonest cause (57.4%) of reporting to the emergency. Psychiatric comorbidity was found among 7.4%. Commonest medications used were chlordiazepoxide and clonidine, for withdrawal and naltrexone, for substitution. No standardized treatment protocol for substitution treatment was available.

**Conclusions:**

This is an initial attempt to study the sociodemographic and drug use profile of substance abusers in Sikkim. Demographic and socioeconomic characteristics of substance abusers seeking emergency services are not significantly different from treatment-seeking substance abusers in other parts of India. IDU behaviour has been detected and low median duration of use suggests an emerging problem and need for urgent harm reduction. Alcohol withdrawal was the commonest cause of seeking emergency services, which is related to high prevalence of alcohol abuse in Sikkim. No standardized substitution treatment is available for substance abusers, which may lead to higher rates of relapse.

## INTRODUCTION

Sikkim, a hilly state in Northeast India and the second smallest state of India, is located in the foothills of the Himalayas and shares international borders with Nepal, Bhutan and Tibet. Sikkim is inhabited by indigenous population of Lepchas, Bhutias and Nepalis. Lepchas are traditional inhabitants of Sikkim, whereas Bhutias and Nepalis have migrated from Tibet and Nepal, respectively. Sikkim has an approximate population of 540,493,^1^ a literacy rate of 70% and a landscape varying from 300 to 8585 metres in altitude.^2^ Sikkim has traditionally been a royal state and was annexed to India in 1975.^3^

From unofficial estimates alcohol use has traditionally been prevalent among Sikkimese population. National Family Health Survey-2, Government of India, has also highlighted a significant prevalence of alcohol use in Sikkim—32% and 17% among above 15 years of age males and females, respectively.^2^ From their observations prevalence of alcohol use is more common in rural areas than urban and negatively related to level of education and socioeconomic condition.^2^ These rough estimates make it obvious that alcohol use has become an important public health issue in Sikkim. Moreover, since its annexure to Indian state, migration of people from other parts of India has increased and has resulted in introduction of other substance abuse practices in the community, which was so far unexposed to external influences, cultures and practices. As a result, abuse of opioids including heroin and other synthetic opioids have been reported from treatment centers. Similarly injection drug use (IDU) behaviour has also been reported. It is important to note in this context that other Northeast Indian provinces, particularly Manipur, are a significant site of IDU behaviour and resulting blood-borne infections including HIV/AIDS.^4^ It is, therefore, obvious that Sikkim is going through a social transition, which is reflected in the changing substance abuse practices in the community. However, to date, any systematic information on epidemiology of drug use behavior in Sikkim is not available.

Emergency services utilization by substance abusers is one of the sources and indicators of assessing problematic substance use. It is an important measure to assess treatment demand from substance abusers and can be an effective tool for a preliminary assessment of magnitude and pattern of substance abuse in the community.^5^ Therefore, studying the profile of substance abusers in the emergency services has been conceived as a tool to have an idea about the nature of substance abuse in the community. This may also help to create a baseline data about substance abuse in Gangtok and nearby areas served by the Central Referral Hospital (CRH). The CRH is the teaching hospital of the Sikkim Manipal Institute of Medical Sciences (SMIMS) and is one of the two tertiary care hospitals in Sikkim providing treatment to substance abusers.

## METHODS

### Patients

Individuals having a history of current substance use and utilizing emergency services at the Central Referral Hospital, SMIMS for any reason related to substance use behaviour were included in the study.

### Inclusion Criteria

1. Current substance user: Substance use for more than 10 days in the past 30 days^5^
2. Seeking treatment for any medical reason related to substance use, as diagnosed by the attending physician
3. Either sex
4. Any age group

### Exclusion Criteria

1. Accidental poisoning
2. Seeking treatment for a medical reason unrelated to substance use, as diagnosed by the attending physician

### Design

The study design was a retrospective chart review. Data were collected retrospectively from July 2000 to June 2005 (60 months). Data sources were emergency case register, hospital records and case sheets.

### Data Collection

Emergency visit records for all patients from July 2000 were reviewed on a monthly basis from the emergency case register with assistance from the emergency medical officer, in charge. The hospital identification number of patients making emergency visits with possible diagnosis of substance use related medical causes were noted separately. After emergency case register was screened up to July 2005, individual hospital records and case sheets of patients having possible substance use related medical cause were accessed for detailed patient and disease related information. The information, thus accessed, was noted in a pre-devised case record form (CRF). The CRF was devised in partial modification and adaptation of the following.

1. Pompidou Group: Core Data for Drug Treatment Reporting System
2. Drug Abuse Warning Network (DAWN) Medical Examiner Report
3. General population Survey of Drug Abuse, WHO, 2000

The CRF contained the following information.

1. Demographic variables, e.g. age, sex, religion, marital status, community, occupation, etc.
2. Socioeconomic variables, e.g. income, education, family information, etc.
3. Drug use variables, e.g. type of drug, duration, route of use, etc.
4. Deviances and high-risk behaviour variables, e.g. property crimes, law infringement, injection sharing, visits to commercial sex workers (CSWs), etc.
5. Reasons for seeking treatment, e.g. overdose, withdrawal, accident, medical consequence, etc.
6. Treatment details and outcome, e.g. diagnosis, medications, previous treatment, complications, disability, death, etc.

### Ethical Issues

The study consisted of only retrospective data analysis from case records and did not involve any patient contact, medical, behavioural, therapeutic or instrumental intervention. The study protocol and CRF were approved by the Institutional Ethical Committee (IEC).

### Data Analysis

Data was cleaned and initially fed in a MS Excel file and later was analyzed using SPSS (Statistical Package for the Social Sciences, SPSS Inc., Chicago, USA) version 10.0.1 using the chi-square test for non-parametric data. Individual risk factors were described by odds ratio with 95% confidence interval. Level of significance was set at p<0.05.

## RESULTS

From July 2000 to June 2005 (60 months), a total of 4648 patients were registered for consultation in the department of psychiatry out of which 227 (4.88%) were diagnosed with substance use disorders. Of these 227 patients with substance use disorder, a total of 54 (23.79%) had their first contact in the emergency. These 54 patients have been included in the current study had been on initial contact attended by the emergency officers, following which specialist referrals were made. If it was an obvious case of substance related problem, e.g. withdrawal, the first specialist referral had been to department of psychiatry, following which the patient got registered in the department of psychiatry. However, if the patient had a substance-related medical/surgical complication, e.g. cellulitis; the first specialist referral had been to department of medicine or surgery, following which a referral was made to department of psychiatry to manage substance use disorder. In either situation, the concerned patient got registered with department of psychiatry.

Table 1 shows the demographic and socioeconomic characteristics of patients with substance use disorders utilizing emergency services at the CRH. It is important to note that gender, history of migration, marital status and community where the person lives have been detected as significant risk factors for emergency visits with alcohol-related disorders.

**Table 1:** Socioeconomic and demographic characteristics of substance abusers utilizing emergency services (*n*=54)

Variable	Category	Percent	*n*	OR	95% CI
Gender	Female	18.5	10	1.375^a^	1.147–1.648^a^
	Male	81.5	44		
Ethnicity	Bhutia	9.3	5	–	–
	Lepcha	5.6	3	–	–
	Nepali	61.1	33	–	–
	Other	24.1	13	–	–
Education	Up to school	16.7	9	–	–
	Up to graduate	46.3	25	–	–
	>Graduate	37.0	20	–	–
Occupation	Agriculture	9.3	5	–	–
	Housewife	11.1	6	–	–
	Salaried	31.5	17	–	–
	Self-employed	25.9	14	–	–
	Student	14.8	8	–	–
	Unemployed	7.4	4	–	–
Marital status	Single	27.8	15	2.846^a^	1.386–5.844^a^
	Married	72.2	39	0.077^b^	0.019–0.311^b^
Community	Rural	37.0	20	1.156^a^	0.880–1.519^a^
	Urban	63.0	34	0.567^b^	0.173–1.852^b^
Religion	Buddhist	22.2	12	–	–
	Christian	3.7	2		
	Hindu	74.1	40	–	–
Migration	Yes	24.1	13	1.348^a^	0.858–2.117^a^
	No	75.9	41	0.444^b^	0.169–1.163^b^

OR=Odds Ratio, 95% CI=95% confidence interval,

a OR for alcohol,

b OR for other drug

Tables 2 and 3 show drug use characteristics by substance abusers attending the emergency room. Median (22 years) of age of first drug use signifies an onset of substance abuse practice among young adults rather than in the school-going age. Cannabis use (*n=*3) as the only drug has been observed among college-going students. Similarly the only primary heroin user (*n*=1) in this group has been a smoker and chaser. Sedatives (nitrazepam) and cough preparations (codeinecontaining) were also used by polydrug abusers (*n*=11). Commonly abused other opioids included dextropropoxyphene and pentazocine and both were used only as injection (IDU). Dextropropoxyphene was injected by emptying contents of a capsule in water and making a suspension. Among the IDUs (*n*=9), the range of duration of drug use is 1–12 years (median 3 years) and the age range is 20–43 years (median 25 years). Out of these 9 IDUs, only 3 consented and underwent voluntary HIV and HBV testing after counselling and all were found seronegative. Five reported needle sharing, three did not volunteer any information and one did not share injection. Out of these 9, 2 gave history of high-risk sex without use of condoms. These 2 also gave a history of needle-sharing and also were tested for HIV and HBV, as described earlier.

**Table 2:** The pattern of drug use among substance abusers utilizing emergency services (*n*=54)

Variable	Range	Median	Mean±SEM
Age (years)	19–68	36	37.77±1.64
Age of first drug use	15–47	22	25.25±1.15
Duration of use	1–35	12	12.07±1.10

SEM = Standard Error of Mean

**Table 3:** The pattern of drug use among substance abusers utilizing emergency services (*n*=54)

Variable	Category	Percent	*n*
Current drug use (past 30 days)	Alcohol	77.8	42
	Cannabis	5.6	3
	Heroin	1.9	1
	Other opioids	14.8	8
Frequency (past 30 days)	3–5 days	1.9	1
	6–9 days	11.1	6
	10–19 days	40.7	22
	>20 days	46.3	25
Current polydrug use	>1 drug	13.0	7
	>2 drugs	7.4	4
Injection drug use	Yes	16.66	9

Table 4 describes relevant medical and treatment related information of substance abusers reporting to the emergency. Withdrawal was the commonest cause (57.4%) of reporting to the emergency and except in one due to heroin, all cases were due to withdrawal from alcohol (*n*=30). Cannabis use was the commonest substance associated with psychosis (*n*=3), although alcohol and other opioids were also associated. Alcohol was also the only drug associated with overdose and adverse reaction (disulfiram-like reaction). On the other hand, IDU was most commonly associated with infection related visits (*n*=6) in the form of abscesses or cellulitis at injection sites. Similarly, patients coming with medical complications, e.g. hypoglycaemia or ketoacidosis were diagnosed with alcohol use disorders. Only 2 patients had associated injury, which could have been due to accident or fall from drug intoxication. Four patients also had psychiatric comorbidity, viz., depression, phobia, personality disorder and schizophrenia.

**Table 4:** Medical and treatment information of substance abusers utilizing emergency services (*n*=54)

Variable	Category	*n*	*(%)*
Reason for attending emergency	Adverse reaction	2	3.7
	Infection	9	16.66
	Other medical	2	3.7
	Overdose	3	5.55
	Psychiatric	7	12.96
	Withdrawal	31	57.4
Associated comorbidity	Injury	2	3.7
	Psychiatric	4	7.4
Primary medication	Chlordiazepoxide	37	68.5
	Clonidine	2	3.7
	Diazepam	5	9.3
	Naltrexone	4	7.4
	Non-specific	5	9.3
Secondary medication	Clonidine	1	1.9
	Diazepam	11	20.4

Chlordiazepoxide was the commonest drug employed for medical treatment and was used in all alcohol-related conditions. In some cases, a combination of chlordiazepoxide and diazepam was also used (*n*=10) for management of alcohol related conditions. Clonidine was employed in treatment of opioid related conditions (*n*=3). Naltrexone was used in two patients with alcohol overdose and as a mode of maintenance treatment in 2 patients with dextropropoxyphene abuse. However, no standardized mode of behavioural and/or maintenance treatment could be made available to the patients. Moreover, no record of follow up treatment was available.

## DISCUSSION

This study is a preliminary attempt to address the pattern of substance abuse in Sikkim, a state in Northeast India. Patients with substance use disorders are significant users of emergency services and inpatient hospital care, arising out of medical or surgical consequences of substance abuse.^6^ Therefore, emergency services utilization provides an indicator of treatment demand from consequences of substance abuse as well as an indirect assessment of trends and patterns of substance abuse in the community.

In the present study an initial significant feature had been a high proportion of female substance abusers seeking treatment in the emergency (18.5%). In the Drug Abuse Monitoring Survey (DAMS), 2002 conducted in 203 treatment centers in different states of India on treatment seeking substance abusers, the percentage of female treatment seeking substance abusers had been 2.8%. However, keeping in mind a significant prevalence of alcohol use in Sikkim—32% and 17% among those above 15 years of age men and women, respectively,^2^ this observation with women substance abusers can be explained. A high percentage of emergency attendance among the Nepalese population (61.1%) in this study can be explained by the fact that the predominant ethnic population in Sikkim is Nepalese.^7^ A high rate of substance abuse among minimum college education completed, employed and married population is also not significantly different from the observations of DAMS, 2002, where 42% of the treatment seekers have completed higher secondary education and above, 70% are employed and 71.9% are married. Therefore, these observations do not vary significantly from other states of India. However, in spite of only 11% urban areas in Sikkim,^2^ there is a disproportionately higher percentage of emergency service users (63%) from urban areas, which also differs from DAMS, 2002 observations (48.3%). This may be explained by the fact that the CRH is located in Gangtok, which is the major urban town of Sikkim and therefore, patients visiting the hospital are more likely to represent urban areas. Hinduism is the predominant religion in Sikkim,^2^ which explains higher percentage of Hindus seeking emergency treatment.

The age range of substance abusing (all drugs) population attending the emergency room is 19 to 68 years with a median of 36 years. However, the age range of IDU population is 20 to 43 years with a median of 25 years. This difference suggests possibility of IDU practice among a younger population, while the older population is using other drugs, mainly alcohol. Similarly, the median of duration of abuse with all drugs is 12 years, while the median with IDU population is 3 years, which also corroborates the earlier conclusion. This also signifies that the IDU practice is a phenomenon of comparatively recent onset. Moreover, dextropropoxyphene and pentazocine are common opioids used for injection with an insignificant use of heroin. This could be suggestive of stricter law enforcement control over narcotic smuggling and as a consequence, increased use of opioids available as prescription medicines. Median of age of first drug use (22 years) signifies an onset of substance use practice among young adults rather than in the school going age, which also corroborates the earlier finding of a higher use among minimum college education completed population. These observations are also not significantly different from DAMS, 2002.^8^

The predominant substance abuse related emergency attendance is with alcohol (77.8%). A significant number of patients (46.3%) have a history of more than 20 days/month frequency of substance abuse. Alcohol use is traditionally prevalent in Sikkim.^2^ However, DAMS, 2002 also observes that the majority of treatment seekers (43.9%) in India abuse alcohol. Moreover, alcohol abuse is a global problem and similar studies describing alcohol related visits to the hospital, as a predominant substance of abuse, has been reported across continents, e.g. in France,^9^ Germany^10^ and USA.^11^ On the other hand, only one Indian study^12^ reported alcohol-related problems making up 17.6% of the caseload of psychiatric emergencies, which is significantly less compared to this study. Moreover, the police brought 75% of them, which varies significantly from the observations in this study, because none of the visiting patients in this study were escorted by the police.

Alcohol withdrawal (57.4%) had been the commonest cause of attending the emergency, which is explicable on the basis of high prevalence of alcohol abuse in this part of India. This study detected psychiatric comorbidity in only 7.4%, which differs from the observations of Adityanjee *et al*.,^12^ where a psychiatric illness was definitely present in 40% of the cases.

This study also highlights the limitation of availability of medications for treatment of substance abuse in this setting. The commonest drug employed for management of alcohol withdrawal had been chlordiazepoxide, a long-acting benzodiazepine. However, there is no standardized protocol of maintenance substitution treatment for alcohol or opioids. Clonidine, an á-2 adrenergic agonist, was employed for opioid withdrawal and naltrexone, an opioid antagonist, was the only drug employed for substitution in four patients with alcohol abuse disorder. Lack of standardized treatment protocol for substance abuse has, thus, resulted in limited follow up visits by the patients and also increases chances of relapse. Adityanjee *et al.,* 1989 has also observed that only 10% of the patients with alcohol related problems were referred for outpatient treatment and 85% were not given any follow up advice.

One of the major limitations of this study is poor quality data availability from hospital case records. However, this is a common problem with retrospective data analysis depending on patient case records. Also, the records of the patients included in the study have been traced from the psychiatry department and thus there is a likelihood of some patients being missed during the process of referral.

In conclusion, this is an early attempt to address the problem of substance abuse in Sikkim and these observations might be helpful in future in designing larger epidemiological investigations.

## CONFLICT OF INTEREST

None
